# The growing challenge of arboviruses in Latin America: Dengue and Oropouche in focus

**DOI:** 10.1371/journal.pntd.0012789

**Published:** 2025-01-07

**Authors:** Joziana Muniz de Paiva Barçante, José Cherem

**Affiliations:** Department of Medicine, Biomedical Research Center (NUPEB), Federal University of Lavras, Lavras, Brazil; University of California Berkeley, UNITED STATES OF AMERICA

In recent years, Latin America has emerged as a critical region on the global epidemic landscape due to the exponential rise in arboviral diseases, including yellow fever, chikungunya, Zika, dengue, and more recently, Oropouche fever. The Zika epidemic of 2015, coupled with the unprecedented increase in dengue cases and the yellow fever outbreak earlier this year, foreshadowed even more complex epidemiological challenges. The years 2020 and, particularly, the first half of 2021 (up to epidemiological week 26) in Latin America and the Caribbean were marked by the COVID-19 pandemic, which had severe impacts on public health. This period clearly illustrates a syndemic scenario, characterized by the interaction between the SARS-CoV-2 pandemic and a significant increase in arboviral diseases, with a total of 3,428,831 cases reported by June 2021. This situation highlights the overlap of a global pandemic with an ongoing endemic of arboviral diseases, resulting in additional challenges for the region’s healthcare systems, which were already overwhelmed by the health crisis caused by COVID-19 [1]. We are now confronted with the expansion of the Oropouche virus (OROV), an emerging arbovirus in South and Central America with a high potential for widespread dissemination [2]. Given the endemic nature of OROV infection in these areas, the US Centers for Disease Control and Prevention has recently elevated Brazil to a level 1 Travel Health Alert [3].

Since the first isolation of OROV, >30 outbreaks have been reported in Brazil, Peru, Panama, and Trinidad and Tobago during 1960–2009, with at least half a million persons estimated to have been infected [4]. OROV was described in Brazil in 1960 when it was isolated from a sloth (*Bradypus tridactylus*) captured near a forested area during the construction of the Belem–Brasilia highway and from a pool of *Ochlerotatus* (*Ochlerotatus*) *serratus* mosquitoes, captured near the same site [5]. Nowadays, OROV is primarily transmitted by *Culicoides paraensis*, commonly found in water bodies such as ponds, lakes, and rivers and humid tropical regions, particularly in some areas of different South American countries, and it plays an important role in the transmission dynamics of OROV [3]. Given the potential role of *Culex quinquefasciatus* in the epidemiology of OROV and the broad geographic range of this species, OROV could also be dispersed by this vector into new geographic areas, including Africa, Australia, and Southeast Asia [6]. Several studies suggest that OROV is expanding beyond endemic regions, positioning itself as a potential candidate for large-scale epidemics [7–9].

OROV, an arbovirus of the Orthobunyavirus genus in the Peribunyaviridae family, has become a growing public health concern in several South and Central American countries. Initially restricted to small Amazonian villages, OROV has now spread to large urban centers in Brazil, with 11,695 confirmed cases by December 2024, across all 27 federal units. [10]. The symptoms are similar to dengue, including acute fever, photophobia, joint and muscle pain, nausea, vomiting, and, in severe cases, hemorrhagic or neurological complications such as diplopia, nystagmus, and meningoencephalitis [11,12]. Approximately 60% of infected individuals experience relapse episodes about 15 days after the initial symptoms resolve.

The infection is also associated with neurological complications, with OROV genomic RNA detected in the cerebrospinal fluid of patients, suggesting its neuroinvasive potential [13]. In May 2024, Cuba reported its first outbreak of OROV, affecting 74 individuals, of whom 3 (4%) developed a clinical diagnosis of Guillain-Barré Syndrome, a severe neurological complication requiring high-complexity supportive care. Guillain-Barré Syndrome is one of the potential neurological complications associated with OROV infection, as evidenced by these cases. This scenario is reminiscent of the Zika virus epidemic, where similar post-viral neurological disorders were observed, emphasizing the need to strengthen healthcare systems in preparation for potential OROV epidemics. Such measures include scaling up intensive care services and rehabilitation facilities to ensure adequate support for affected patients and to mitigate the burden of this emerging infectious disease [14]. This year Brazil has seen more than 11 times as many cases of the virus than in 2023. A significant challenge for maternal-fetal health is the Brazilian scientists’ report indicating that OROV, on the rise in South and Central America, may cause stillbirths and neurological defects in fetuses infected in utero [15,16]. The virus was detected in the umbilical cord blood and tissue samples from several key organs obtained from a stillborn baby, who was 30 weeks into pregnancy, and later tested positive for the virus. These cases evoke memories of similar issues during the Zika outbreaks in the Americas. Over 3,500 cases of infant microcephaly were reported during the major Zika outbreak in Brazil in 2015 and 2016, when it was estimated that 1.5 million people were infected [15].

Another alarming signal was the confirmation of the first deaths related to OROV, marking a significant milestone in the history of this arboviral disease. On July 25, the Brazilian Ministry of Health confirmed the first scientific evidence of deaths caused by OROV: two women in their thirties, without comorbidities, from the state of Bahia, who developed fatal complications following infection. A third case, in the state of Santa Catarina, is still under investigation. These unprecedented mortality records since the identification of OROV in 1955, coupled with the risk of fetal malformations and miscarriages, underscore the urgent need for a national genomic surveillance center to monitor the virus and its mutations, aiming to understand changes in the epidemiological profile of the disease and mitigate future risks.

Like dengue, the OROV exhibits significant genetic diversity, with four distinct genotypes. All four OROV lineages have been found in Brazil, suggesting a plausible origin of the virus or high overlap in the distribution of vectors and virus reservoirs [4]. This fact makes genomic surveillance an indispensable tool for monitoring viral evolution, identifying mutations that may impact transmissibility, virulence, and immune response, and guiding control strategies and vaccine development.

The advancement of the OROV epidemic, alongside recurrent dengue epidemics throughout Latin America, signals significant failures in the control efforts for these diseases. In 2024, dengue reached alarming numbers, with 12,820,082 reported suspected cases throughout the 12 months of the year a historic record according to the Pan American Health Organization. Brazil leads this unfortunate ranking, with 10,099,260 suspected cases and over 5,925 deaths, representing 75.43% of the fatalities in the Americas Region [17]. All four dengue virus serotypes (DENV-1, DENV-2, DENV-3, and DENV-4) are in circulation, increasing the risk of even more severe epidemics due to the possibility of successive infections [17].

The reasons behind the increase in arboviral cases, especially in the Americas, are complex and multifactorial. Climate changes, such as intense heat waves and alterations in rainfall patterns are contributing to the expansion of arboviruses and the populations of the insects that vector them. Additionally, unplanned urbanization and lack of adequate infrastructure in vulnerable areas hinder control measures, exacerbating the situation [18].

The rise in arboviral diseases in Brazil highlights the urgent need for a robust and integrated epidemiological surveillance system. Furthermore, the increasing number of Oropouche fever cases may be just the tip of the iceberg [19]. It is essential to conduct entomological research to clarify the role of each vector, strategies to control these vectors, and the non-vectorial modes of OROV transmission. Many dengue case reports are based on the clinical–epidemiological criteria due to the lack of confirmatory laboratory diagnosis. Given the symptom similarity between dengue and Oropouche fever, we may be facing hundreds or thousands of cases with misdiagnoses. This underscores the necessity of establishing a national epidemic surveillance center, including genomic surveillance and data integration across all Brazilian states, which is crucial for controlling these diseases and protecting public health. Such a center would enable early detection and rapid response to emerging outbreaks, which is essential given the evident shortcomings in current arboviral surveillance and control efforts. It is imperative that we mobilize global resources and efforts to identify the best approaches to effectively and sustainably combat these threats. As environmental changes create favorable conditions, vector insects expand their geographical distribution, and problems currently concentrated in the Americas may reach global proportions without coordinated international action.
